# Unusual metabolic characteristics in skeletal muscles of transgenic rabbits for human lipoprotein lipase

**DOI:** 10.1186/1476-511X-3-27

**Published:** 2004-12-09

**Authors:** Florence Gondret, Sanjay B Jadhao, Marie Damon, Patrick Herpin, Céline Viglietta, Louis-Marie Houdebine, Jean-François Hocquette

**Affiliations:** 1 INRA, UMR sur le Veau et le Porc, 35590 Saint Gilles, France; 2 INRA, Unité de Recherche sur les Herbivores, 63122 Saint-Genès Champanelle, France; 3 INRA, Biologie du Développement et Reproduction, Domaine de Vilvert, 78352 Jouy-en-Josas cedex, France

## Abstract

**Background:**

The lipoprotein lipase (LPL) hydrolyses circulating triacylglycerol-rich lipoproteins. Thereby, LPL acts as a metabolic gate-keeper for fatty acids partitioning between adipose tissue for storage and skeletal muscle primarily for energy use. Transgenic mice that markedly over-express LPL exclusively in muscle, show increases not only in LPL activity, but also in oxidative enzyme activities and in number of mitochondria, together with an impaired glucose tolerance. However, the role of LPL in intracellular nutrient pathways remains uncertain. To examine differences in muscle nutrient uptake and fatty acid oxidative pattern, transgenic rabbits harboring a DNA fragment of the human LPL gene (hLPL) and their wild-type littermates were compared for two muscles of different metabolic type, and for perirenal fat.

**Results:**

Analyses of skeletal muscles and adipose tissue showed the expression of the hLPL DNA fragment in tissues of the hLPL group only. Unexpectedly, the activity level of LPL in both tissues was similar in the two groups. Nevertheless, mitochondrial fatty acid oxidation rate, measured *ex vivo *using [1-^14^C]oleate as substrate, was lower in hLPL rabbits than in wild-type rabbits for the two muscles under study. Both insulin-sensitive glucose transporter GLUT4 and muscle fatty acid binding protein (H-FABP) contents were higher in hLPL rabbits than in wild-type littermates for the pure oxidative *semimembranosus proprius *muscle, but differences between groups did not reach significance when considering the fast-twitch glycolytic *longissimus *muscle. Variations in both glucose uptake potential, intra-cytoplasmic binding of fatty acids, and lipid oxidation rate observed in hLPL rabbits compared with their wild-type littermates, were not followed by any modifications in tissue lipid content, body fat, and plasma levels in energy-yielding metabolites.

**Conclusions:**

Expression of intracellular binding proteins for both fatty acids and glucose, and their following oxidation rates in skeletal muscles of hLPL rabbits were not fully consistent with the physiology rules. The modifications observed in muscle metabolic properties might not be directly associated with any LPL-linked pathways, but resulted likely of transgene random insertion into rabbit organism close to any regulatory genes. Our findings enlighten the risks for undesirable phenotypic modifications in micro-injected animals and difficulties of biotechnology in mammals larger than mice.

## Background

The endothelial cell-associated lipoprotein lipase (LPL) works to break down triacylglycerol-rich dietary fats absorbed after a meal, thus generating free fatty acids transported in the blood. Earlier works suggested that LPL acts as a metabolic gate-keeper for fatty acid partitioning between adipose tissue for storage and muscle primarily for energy use [1]. Then, variation of LPL activity among fat depots as well as ratio of adipose tissue to skeletal muscle LPL activity, have been proposed to be linked to the development of regional obesity under certain genetic predisposition [2]. Transgenic mouse lines that highly over-express LPL exclusively in muscles, further evidence a role of LPL in the intracellular fate of nutrients into skeletal muscles. Indeed, induced mutant mice over-expressing human LPL (hLPL) exclusively in muscles have, proportional to the level of LPL transgene expression, increases in LPL activity and free FA concentration in muscle [3], a higher number of metabolic organelles (mitochondria, peroxisomes) in muscles [4], and elevated muscle oxidative enzymes activities [3,4]. Thus, a greater use of lipids for energy production during fasting has been suggested in transgenic hLPL mice [5]. In agreement with the inverse relative rates of fatty acid oxidation and glucose utilization in muscle first proposed by Randle and coworkers [6], mice with over-expression of hLPL specifically in muscles show alterations in muscle glucose metabolism, such as elevated blood glucose levels [7], increased glycogen stores [3] or glucose-6-phosphate content [5], and(or) impaired glucose tolerance [5,8]. Finally, as shown in mice over-expressing a mutant defective hLPL, enhanced lipoprotein uptake into cells may also occur via pathways independent of LPL catalytic activity, resulting in a mitochondriopathy as well as in muscle glycogen accumulation similar to the pattern observed in mice expressing active hLPL [9]. However, another point not studied so far in hLPL transgenic animals is that uptake of nutrients and(or) intra-myocellular trafficking to target organelles are facilitated to a great extent by specific transporters and(or) binding proteins. Convincing data are available for the involvement of both membrane-associated and cytoplasmic fatty acid-binding proteins in fatty acid uptake by skeletal muscles [10,11]. Especially, a permissive action of heart-type fatty acid binding protein (H-FABP), also known as muscle FABP or FABP3, in delivering fatty acids to mitochondrial β-oxidation systems has been shown [12,13]. Facilitated glucose transport across membranes of muscle cells mediated by GLUT4 is usually considered as rate-limiting for glucose utilization by skeletal muscles in laboratory rodents [14]. However, it remains to determine whether LPL effects on muscle oxidative pathways involve modifications in the intracellular binding of nutrients.

Altogether, metabolic studies in muscles of transgenic animals help to understand the biological links between fatty acid uptake, intracellular lipid metabolism, and some metabolic disorders such as diabetes in human beings. However, most data have been established in mice. Potential advantages of rabbit compared with mouse as human disease model [15,16] relate in part to its lipoprotein profile which more closely mimics that of humans. Therefore, this study aimed to characterize the oxidative phenotype of two skeletal muscles in transgenic rabbits harboring a DNA fragment of the human LPL gene. The study revealed that despite lack of differences in tissue LPL activity when compared with their wild-type littermates, transgenic hLPL rabbits displayed modest increases in both H-FABP and GLUT4 contents in a pure oxidative muscle and significant lower mitochondrial fatty acid oxidation rates in two skeletal muscles differing in their fiber type composition. This suggested that a random insertion of hLPL DNA into the rabbit genome resulted into unexpected disruption of target nutrient pathways.

## Results

### Tissue LPL level

**Figure 1:**
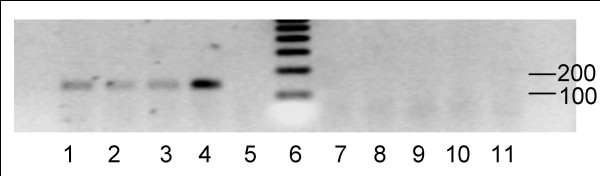
**Expression of human lipoprotein lipase (hLPL) mRNA in transgenic rabbits. **The cDNA obtained by reverse transcription (RT) of total RNA extracted from skeletal muscles or perirenal fat and primed by random primers followed by 35 cycles of PCR with hLPL-specific primers, was loaded on 2% agarose gel. Reaction was performed in parallel in the absence of reverse transcriptase (RT-), to ensure for lack of genomic DNA contamination. Typical RT-PCR results are shown for *semimembranosus proprius *muscle. Lanes 1–4: RT-PCR product in hLPL rabbit; Lane 5: RT- in hLPL rabbit; Lane 6: 100 bp DNA ladder; Lane 7–10: RT-PCR product in wild-type rabbit; Lane 11: RT- in wild-type rabbit. A band at the expected size of 137 bp was detected in hLPL rabbits only.

An amplification product corresponding to hLPL fragment was evidenced in adipose tissue, *semimembranosus proprius *and *longissimus *muscles of hLPL group, proving the expression of the transgene in tissues of the rabbit organism. On the contrary, no signal was detected in the same tissues of wild-type animals (figure 1). Surprisingly, hLPL rabbits and their wild-type littermates exhibited similar LPL activity for adipose tissue and muscles (table 1).

**Table 1:** Lipoprotein lipase activity^1 ^in tissues of wild-type and hLPL transgenic rabbits

Tissues	Wild-type rabbits	hLPL rabbits
Perirenal fat	1305 ± 336	1420 ± 500
SMP muscle	2036 ± 583	1546 ± 397
LL muscle	493 ± 111	396 ± 58

^1^Activities are presented as mean ± SEM in perirenal fat, *semimembranosus proprius *(SMP) and *longissimus *(LL) muscles (nmol free fatty acids released. min^-1 ^per g of tissue). There were no differences among groups (*P *> 0.10).

### Plasma metabolites and tissue lipids

Plasma concentrations in triglycerides, free fatty acids, and glucose, were similar in the two genetic groups (table 2). Moreover, there were no differences between the two groups for lipid contents in muscles and perirenal adipose tissue (table 3), as well as for fat proportion relative to body weight (18.8 ± 1.0 g/kg and 20.6 ± 0.9 g/kg in wild-type and hLPL rabbits, respectively).

**Table 2:** Plasma triglycerides, free fatty acids and glucose levels^1 ^in wild-type and hLPL transgenic rabbits

Plasma metabolites	Wild-type rabbits	hLPL rabbits
Triglycerides	0.69 ± 0.13	0.81 ± 0.14
Free fatty acids	0.41 ± 0.10	0.41 ± 0.11
Glucose	9.21 ± 0.60	8.60 ± 0.27

^1^Concentrations are presented as mean + SEM (mmol/L). There were no differences among groups (*P *> 0.10).

**Table 3:** Lipids and intracellular nutrient trafficking^1 ^in perirenal fat and skeletal muscles in wild-type and hLPL rabbits

	Wild-type rabbits	hLPL rabbits
Perirenal fat		
Lipids	677 ± 25	691 ± 31
GLUT4	198 ± 18	248 ± 30
*Semimembranosus proprius *muscle		
Lipids	46.5 ± 4.8	42.9 ± 6.5
H-FABP	222 ± 19	*289 ± 20
GLUT4	96.8 ± 14.9	†131.4 ± 8.5
*Longissimus *muscle		
Lipids	13.1 ± 2.3	11.8 ± 1.3
H-FABP	20.4 ± 4.6	32.0 ± 6.0
GLUT4	76.3 ± 11.8	94.4 ± 12.2

^1^Data are presented as mean + SEM in perirenal fat, *semimembranosus proprius *(SMP) and *longissimus *(LL) muscles. Abbreviation used: au (arbitrary units).

*Difference in heart-fatty acid bind protein content (H-FABP) in hLPL rabbits as compared with wild-type littermates (*P *< 0.05).

†Difference in insulin-sensitive glucose transporter GLUT4 in hLPL rabbits as compared with wild-type littermates (*P *< 0.10).

### Nutrient oxidative pathways

**Figure 2:**
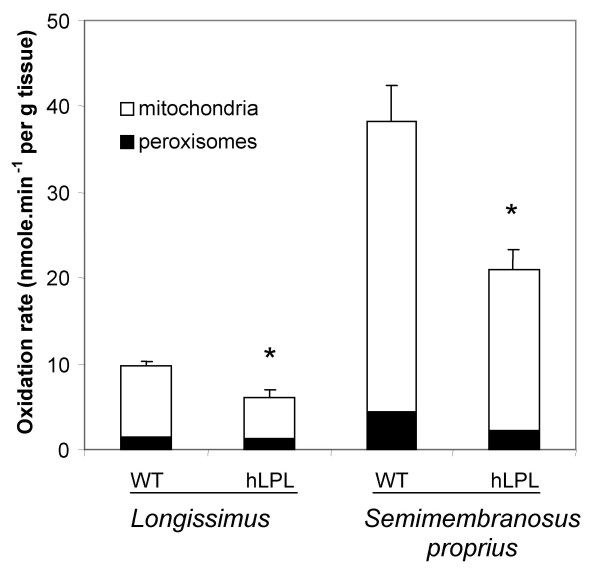
**Mitochondrial and peroxisomal oxidation rates of oleate. **Oxidation rates were measured in freshly excised samples of *semimembranosus proprius *and *longissimus *muscles, using [1-^14^C]oleate as substrate in the presence (peroxisomal oxidation) or absence (total oxidation) of mitochondrial inhibitors. Mitochondrial oxidation rates were calculated by difference between total and peroxisomal oxidation rates. Values shown are mean ± SEM of oleate oxidation (nmole/min^-1 ^per g of muscle wet weight). The * indicates a significant difference (*P *< 0.05) in mitochondrial oxidation rate in hLPL rabbits in comparison with their wild-type (WT) littermates.

Muscle content in H-FABP (responsible for cytoplasmic binding of fatty acids in muscle cells), was 30% higher in *semimembranosus proprius *samples of hLPL rabbits compared with wild-type littermates (table 3). The difference between the two groups (+56%) did not reach significance level in the *longissimus *muscle. This was probably related to a high intra-assay variability, due to the low expression of H-FABP in low-fat glycolytic muscles. The content in insulin-sensitive glucose transporter GLUT4 (the first step in glucose utilization by tissues) was 36% higher (*P *= 0.07) in hLPL rabbits than in wild-type animals for *semimembranosus proprius *muscle, but it did not vary in other sites under study (table 3). The mitochondrial oxidation rates of oleate were reduced by 45% and 41% (*P *< 0.05) in *semimembranosus proprius *and *longissimus *muscles, respectively (figure 2), in hLPL rabbits compared with wild-type littermates. By contrast, oxidation rate in peroxisomes did not vary between groups.

## Discussion

Despite a clear evidence for expression of the hLPL transgene in the tissues under study in the hLPL rabbits only, total LPL (human +native) activity in skeletal muscles or perirenal adipose tissue was similar in hLPL rabbits and in their wild-type littermates. This situation contrasts with the moderately enhanced LPL activity in post-heparin plasma reported in another line of hLPL transgenic rabbits [17], and with the marked elevation of LPL activity observed in adipose tissue [18] or skeletal muscle [3,4,7] of hLPL mice. A preliminary study in the heart of our hLPL trangenic rabbits and their wild-type littermates using polyclonal [19] and monoclonal [20] antibodies which recognize different epitopes of the LPL molecule, did not evidence any significant differences in total LPL protein content between groups with both antibodies (data not shown). Furthermore, plasma triglyceride concentration was currently found similar in hLPL transgenic rabbits and wild-type animals, which is again in favor to a similar content in LPL protein among groups rather than to a catalytically defective hLPL enzyme in transgenic rabbits. Indeed, triglyceridemia is consistently found lower in transgenic animals over-expressing a catalytic active hLPL [7,17] and mutant catalytic defective enzyme [9], although this effect may be less pronounced on some genetic backgrounds [9]. Then, a possible explanation is that failure in hLPL mRNA traduction currently resulted in no hLPL protein, due to lack of regulatory essential elements in the transgene sequence [21]. A second explanation may be that expression of hLPL in transgenic animals led to a down regulation of native LPL.

Surprisingly, despite the lack of difference in LPL activity, many metabolic differences were found between hLPL rabbits and their wild-type littermates in *semimembranosus proprius *(a muscle composed exclusively of slow-twitch type I fibers), and to a lesser extent in the fast-twitch glycoytic *longissimus *muscle. However, unlike results in mice, our findings reported a lower muscle fatty acid oxidation rates in hLPL rabbits. Others have observed that over-expression of hLPL gene specifically in skeletal muscle of transgenic mice rather led to a dose-dependent increase in oxidative enzymes and to a proliferation of the oxidative specialized organelles [3,4].

According to the well-known fatty acid-glucose cycle in skeletal muscles [6], decreased fat oxidation is generally associated with increased glucose utilization. In accordance with this rule, the muscle content in insulin-sensitive glucose transporter GLUT4, i.e., the first step of glucose utilization in skeletal muscle [14], was currently found higher in the pure oxidative muscle of hLPL rabbits compared with their wild-type littermates. However, possibly enhanced utilization of glucose was not followed by any variations in blood glucose level. Either elevated blood level [7] or similar plasma concentration in glucose [3] have been observed in hLPL mice compared with wild-type animals.

Finally, the reason for a higher content of intra-cytoplasmic fatty acid binding proteins (H-FABP) in *semimembranosus proprius *muscle of our hLPL rabbits compared with wild-type animals remains largely unknown. Indeed, a preferential involvement of H-FABP in delivering intracellular fatty acids to sites of oxidation has been widely suggested [22]. Here, fatty acid oxidation rate was decreased in hLPL rabbits compared with wild-type littermates, but muscle lipid content and body fat did not vary among groups. One hypothesis may be that fatty acids bound to H-FABP within cell cytoplasm would by-pass any muscle metabolic pathways. If true, fatty acids would be immediately re-exported into the blood circulation and subsequently oxidized in the liver, presumably to prevent muscle from toxicity due to increased fatty acids entry. *In vitro *and *ex vivo *findings indeed recently suggest that non-adipose tissue, such as cardiomyocytes, can re-export fatty acids when influx exceeds oxidation rate [23]. However, there is no clear evidence for such a mechanism in our hLPL rabbits, since plasma concentration of free fatty acids was found similar in the two genetic groups. Various results are reported in the literature data on free fatty acids concentration in serum of hLPL transgenic mammals, with either similar, increased, or decreased levels [3,4] depending of mice strain and level of hLPL over-expression.

Altogether, the various metabolic disruptions in skeletal muscles of hLPL rabbits are in favor to a random integration of the micro-injected hLPL DNA within or close to endogenous genes. In transgenic mice, estimates of the frequency of these insertional mutations range from 7 to 20% [24]. This may have resulted in a loss of function of neighboring genes, aberrant expression patterns, and therefore in unexpected phenotypes. However, genes coding for LPL, H-FABP, GLUT4, and oxidative pathway (e.g. carnitine palmitoyl-transferase I) are not clustered on the same chromosome in the human genome [25] and likely, although not available, in the rabbit map. Therefore, if any, the integration site of the foreign DNA must have conflicted with regulatory elements of any molecular factors able to modify whole nutrient metabolic cascade.

## Conclusions

During the last 15 years, transgenesis has been extended from mice to larger mammals, with the aim of benefiting human health. Transgenic rabbits for LPL gene may offer useful models to test the relationships between uptake of nutrients, intracellular trafficking and subsequent metabolic fate in a species sharing a lipoprotein profile closely similar to that in Human. However, we currently reported alterations of nutrient bindings and oxidative metabolism in skeletal muscles of hLPL rabbits, despite the lack of difference in tissue LPL activity between transgenic rabbits and their wild-type littermates. It is thus suggested that hLPL phenotype emerged from insertional mutation of hLPL DNA within or close to endogenous genes. This study underlined the risk of unpredictable phenotypic properties in micro-injected transgenic rabbits, and thereby the difficulty of animal biotechnology in mammals larger than mice. Nevertheless, transgenic rabbits remain useful tools for understanding the relative importance of the various metabolic pathways involved in the control of tissue lipid content, especially when the genetic map now under progress will be available in the rabbit.

## Methods

### Rabbits

The Genetic Committee of the French Ministry of Agriculture approved the experiment. Rabbits were reared and killed in accordance with the French regulations for humane care and use of animals in research. New-Zealand White rabbit does were super-ovulated by injections of porcine-follicle-stimulating hormone, as previously described [26], and were mated to males of the same genetic background. Embryos were collected 17 hours later. The human LPL (hLPL) fragment of genomic DNA, inserted into a 90-kilobase P1 phagemid together with regulatory elements, was kindly provided by N. Duverger (Aventis, Evry, France). The expression of hLPL fragment in the host organism was governed by the P1 phagemid promoter. DNA solution was injected into the male pronuclei, and the injected embryos were transferred to the pseudo-pregnant females (INRA, Laboratoire de Biologie Cellulaire, Jouy-en-Josas, France). Genomic DNA was extracted from ear biopsy in the offspring [27]. Presence or absence of hLPL DNA was screened by PCR using 5'-CCCTTTTTCCTGTCTTTTT-3' as sense and 5'-AGTGCTTGAGACTGTC-TCCTAA-3' as anti-sense primers. These primers framed a fragment of 201 bp of the human LPL gene spanning intron 9 and exon 10.

Two transgenic littermate male founders were cross-bred with 20 females of a standard New-Zealand White line (A-1067, INRA, France) at the INRA experimental unit (Le Magneraud, Surgères, France), to provide F1 animals for analysis. Pup genotypes were determined at the hLPL locus by PCR from tail tip DNA at the age of 4 weeks, using the primers described above. Control PCR amplification of the actin gene was performed in parallel to ensure DNA quality.

After weaning (5 weeks), young rabbits were housed collectively by genotype (8 animals per cage), under a controlled light/dark cycle (16/8 h). They were offered free access to water and to a standard rabbit pelleted diet (16.5% crude protein, 16.4% cellulose, 2.8% fat, 8.3% ash, and 3790 kcal/kg gross energy). At 10 weeks of age, pairs of hLPL rabbits and wild-type littermates of similar body weight (2400 g ± 53, *n *= 6 in each genotype) were selected within litters, and bled in the fed state.

### Analysis of plasma metabolites

Enzymatic methods adapted to a Cobas Mira multi-analyzer apparatus (ABX, Montpellier, France), were used to determine levels of triglycerides (kit PAP 150, BioMérieux, Marcy l'Etoile, France), free fatty acids (kit Wako NEFA-C kit, Richmond, VA, USA) and glucose (kit PAP 1200, BioMérieux, Marcy l'Etoile, France) in rabbit plasma collected at the time of the death.

### Tissue preparation

Portions of perirenal fat, *semimembranosus proprius *(SMP) as a muscle composed solely of slow-twitch oxidative fibers, and *longissimus *(LL) muscle representing predominantly fast-twitch glycolytic fibers, were stored at -70°C until RNA analysis and biochemical measurements. About 300 mg of each muscle was homogenized immediately after sacrifice in an appropriated buffer for measurements of *ex vivo *oxidation rates, as described previously [28,29].

### RNA analysis for expression of the transgene

Total RNA from 600 mg of tissues was extracted by acid guanidium thiocyanate-phenol chloroform method [30], and was reverse transcribed into cDNA using pd(N)6 random primers (Amersham Biosciences, Orsay, France). Nested PCR (Quiagen, Courtaboeuf, France) was carried out (35 cycles) using hLPL cDNA specific primers, as follows. 5'-TTCTGTGAAGAATGAAGTGG-3' as sense and 5'-AGTGCTTGACA-CTGTCTCCTAA-3' as anti-sense primers framed a 137 bp fragment in the exon 10 of hLPL gene. PCR products were loaded on 2% agarose gel. The amplified fragment was picked up and sequenced (ESGS Cybergene, Evry, France). In each sample, the absence of genomic DNA contamination was checked by performing RT-PCR reaction without reverse transcriptase.

### Lipoprotein lipase activity

Lipoprotein lipase (LPL) activity was assessed after homogenization of the tissues in a buffer composed of ammonia-HCl (25 mM) pH 8.2, containing EDTA (5 mM), Triton-X-100 (8 g/l), sodium dodecyl sulfate (0.10 g/l), heparin (5000 IU/l) and peptidase inhibitors. Insoluble material was discarded by centrifugation at 20000 × *g *for 20 min at 4°C. As previously described [31], rat serum was used as activator, and Intralipid^® ^(Pharmacia, Stockholm, Sweden), into which [^3^H] triolein has been incorporated, was used as the substrate. Liberated [^3^H]-free fatty acids were quantified by liquid scintillation.

### Biochemical analyses

Tissue lipid content was determined after chloroform/methanol extraction [32]. Muscle content in H-FABP was determined by ELISA analysis on cytosolic protein preparations [33] using a rat polyclonal antibody [34]. Taken into account yields of proteins in cytosolic fractions, results were converted into arbitrary densitometric (DO) units per g tissue wet weight. Insulin-sensitive glucose transporter GLUT4 content was investigated by Western-blot analysis [35] using a polyclonal antibody raised against a synthetic peptide of the C-terminal part of GLUT4, on tissue preparations obtained for LPL activity determination. Results were converted into arbitrary densitometric (DO) per g tissue wet weight, after taking into account yields of proteins in tissue preparations.

### Rates of fatty acid oxidation

Oxidation rate of oleic acid was determined in freshly excised muscles as described earlier for rat and bovine muscles [28], with minor modifications to take into account rabbit specificity [29]. Briefly, samples were minced with scissors and homogenized at a tissue concentration of 60 mg/mL in 0.25 M sucrose, 2 mM EDTA, and 10 mM Tris-HCl ice-cold buffer (pH = 7.4), using a glass-glass homogenizer. A tracer amount of [1-^14^C]oleic acid bound to defatted albumin in a 5:1 molar ratio was used as substrate. Oleate oxidation was measured using L-carnitine and other cofactors, in the absence (total oxidation rate) or presence (peroxisomal oxidation) of mitochondrial inhibitors of the respiratory chain (i.e., 75.6 μM antimycin A, and 10 μM rotenone, SIGMA, St-Louis, MO). The difference between total oxidation and peroxisomal oxidation was considered to be mitochondrial oxidation. All assays were performed in triplicates.

### Statistics

The Kruskal-Wallis non-parametric test was used to analyze differences between groups (SAS Inst, Cary NC, NY, USA). All data are presented as mean ± SEM.

## Authors' contributions

FG, JFH and PH conceived of the study, participated in its design and co-ordination. FG, SBJ and JFH carried out biochemical analyses. MD carried out pup genotyping. CV and LMH carried out micro-injection and provide transgenic breeder animals. FG, JFH and MD drafted the manuscript. All authors read and approved the final manuscript.
